# β‐Sitosterol suppresses hepatocellular carcinoma growth and metastasis via FOXM1‐regulated Wnt/β‐catenin pathway

**DOI:** 10.1111/jcmm.18072

**Published:** 2023-12-08

**Authors:** Yuankun Chen, Yijun Yang, Nengyi Wang, Rui Liu, Qiuping Wu, Hua Pei, Wenting Li

**Affiliations:** ^1^ Department of Infectious and Tropical Diseases The Second Affiliated Hospital of Hainan Medical University Haikou Hainan China; ^2^ Key Laboratory of Tropical Translational Medicine of Ministry of Health Hainan Medical University Haikou Hainan China; ^3^ Department of Clinical Laboratory The Second Affiliated Hospital of Hainan Medical University Haikou Hainan China; ^4^ Department of Infectious Diseases The First Affiliated Hospital of Anhui Medical University Hefei Anhui China

**Keywords:** forkhead box M1, hepatocellular carcinoma, metastasis, proliferation, β‐sitosterol

## Abstract

β‐Sitosterol is a natural compound with demonstrated anti‐cancer properties against various cancers. However, its effects on hepatocellular carcinoma (HCC) and the underlying mechanisms are not well understood. This study aims to investigate the impact of β‐sitosterol on HCC. In this study, we investigated the effects of β‐sitosterol on HCC tumour growth and metastasis using a xenograft mouse model and a range of molecular analyses, including bioinformatics, real‐time PCR, western blotting, lentivirus transfection, CCK8, scratch and transwell assays. The results found that β‐sitosterol significantly inhibits HepG2 cell proliferation, migration and invasion both in vitro and in vivo. Bioinformatics analysis identifies forkhead box M1 (FOXM1) as a potential target for β‐sitosterol in HCC treatment. FOXM1 is upregulated in HCC tissues and cell lines, correlating with poor prognosis in patients. β‐Sitosterol downregulates FOXM1 expression in vitro and in vivo. FOXM1 overexpression mitigates β‐sitosterol's inhibitory effects on HepG2 cells. Additionally, β‐sitosterol suppresses epithelial–mesenchymal transition (EMT) in HepG2 cells, while FOXM1 overexpression promotes EMT. Mechanistically, β‐sitosterol inhibits Wnt/β‐catenin signalling by downregulating FOXM1, regulating target gene transcription related to HepG2 cell proliferation and metastasis. β‐Sitosterol shows promising potential as a therapeutic candidate for inhibiting HCC growth and metastasis through FOXM1 downregulation and Wnt/β‐catenin signalling inhibition.

## 1 INTRODUCTION

Hepatocellular carcinoma (HCC), the primary form of hepatic carcinoma and a prevalent malignant tumour, is associated with a poor prognosis.^1^ Various risk factors, such as cirrhosis, autoimmune hepatitis, HBV or HCV infection, nonalcoholic fatty liver disease and alcohol abuse, contribute to HCC progression.^2^, ^3^, ^4^ The aggressive proliferation, metastatic capacity and high drug resistance rates of HCC cells are major factors contributing to their invasive behaviour.^5^, ^6^ Despite significant progress in clinical HCC treatment, the prognosis for patients remains grim due to rapid progression and early recurrence.^7^, ^8^, ^9^ Thus, it is crucial to develop new drugs and identify novel therapeutic targets for HCC treatment.

Forkhead box proteins constitute an evolutionarily conserved family of transcriptional regulators. Among them, forkhead box M1 (FOXM1) is known to be expressed in proliferating cells and associated with the activation of the mitotic programme.^10^ FOXM1 plays a crucial role in cancer progression, influencing various steps such as epithelial–mesenchymal transition (EMT), cell proliferation, migration and premetastatic niche formation.^11^, ^12^, ^13^ In the context of HCC, FOXM1 has been found to be upregulated and contributes to the progression of HCC by directly regulating the expression of KIF4A.^14^ Notably, carfilzomib has demonstrated effectiveness in inhibiting FOXM1 expression in AFP‐positive HCC cells, leading to the inhibition of tumour growth.^15^ These findings underscore the significance of FOXM1 as a potential therapeutic target in HCC treatment.

The canonical Wnt/β‐catenin signalling pathway plays a pivotal role in regulating cell growth, differentiation and migration.^16^, ^17^ Central to this pathway is the β‐catenin protein, which, upon translocation into the nucleus, interacts with the TCF/LEF‐1 transcription factor complex to activate the expression of Wnt target genes, including C‐myc, CyclinD1, MMP2, MMP7 and MMP9.^18^ Dysregulation of this signalling cascade contributes to tumorigenesis and metastasis.

β‐Sitosterol, one of the most prevalent phytosterols, is widely distributed in vegetable oils, nuts and traditional Chinese medicinal plants.^19^, ^20^ Numerous studies suggest that β‐sitosterol hinders the malignant behaviour of cancer cells by inhibiting their proliferation and migration, inducing apoptosis and interfering with cellular metabolism.^21^, ^22^ Despite the significant role of β‐sitosterol in various diseases, its impact on HCC and the underlying mechanism remains unexplored. Thus, the objective of this study was to investigate the inhibitory effect of β‐sitosterol on HCC progression by regulating the FOXM1‐mediated Wnt/β‐catenin signalling pathway.

## 2 MATERIALS AND METHODS

### 2.1 Identifying potential targets of β‐sitosterol against HCC

**FIGURE 1:**
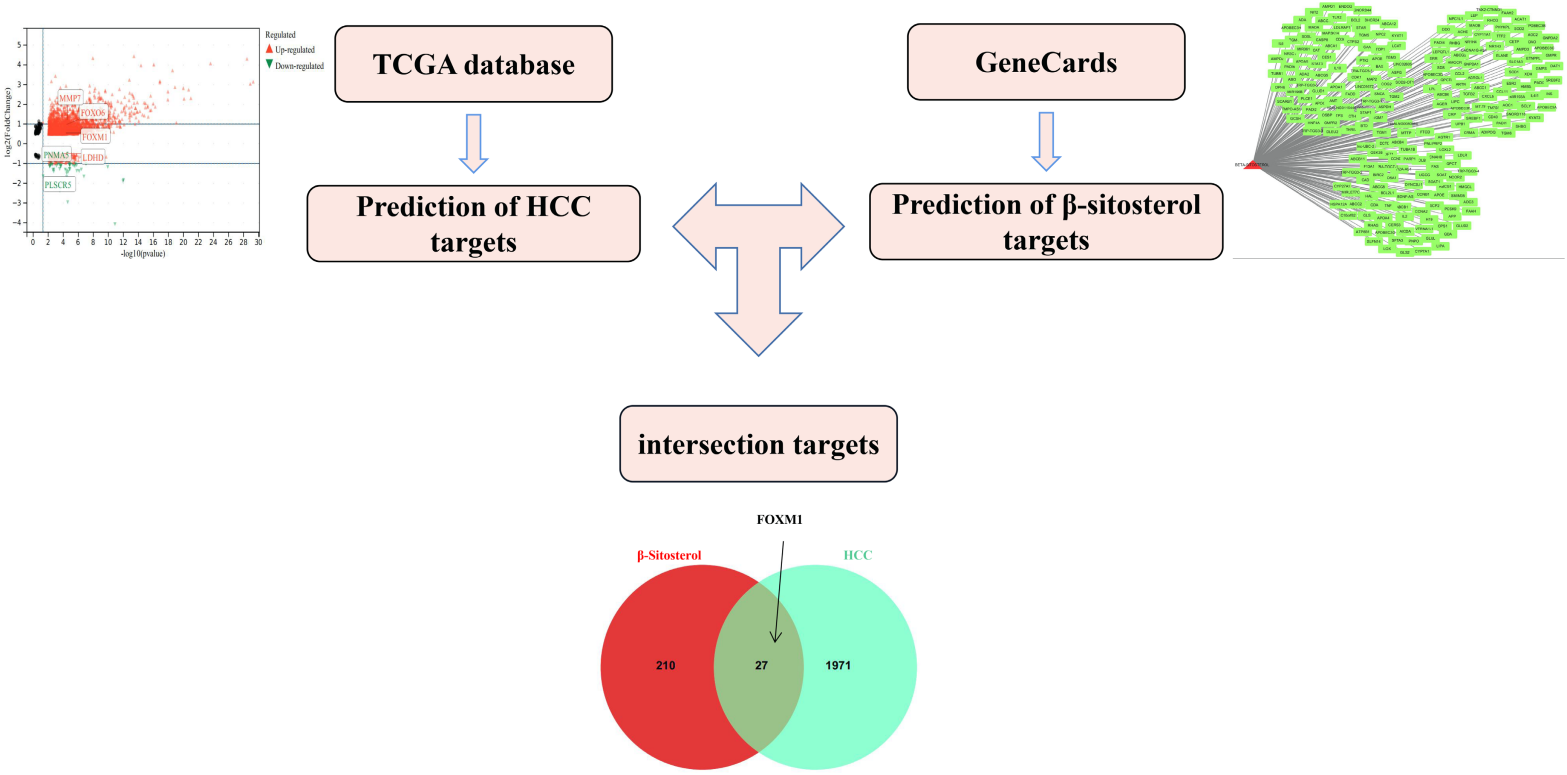
Workflow for predicting potential targets of β‐sitosterol against HCC.

β‐Sitosterol's potential targets were identified through the human gene database (GeneCards, https://www.genecards.org/), while potential targets specific to HCC were obtained from the cancer genome atlas database (TCGA, https://portal.gdc.cancer.gov/). Utilizing the BioLadder bioinformatics online analysis visualization cloud platform (https://www.bioladder.cn/web/#/pro/cloud), a Venn diagram was generated for data comparison. The potential target of β‐sitosterol against HCC was determined by identifying the overlapping genes between its own potential targets and those associated with HCC. The workflow is illustrated in Figure 1.

### 2.2 Retrieval and analysis of FOXM1 expression from databases

To assess FOXM1 expression across different cancer types and normal tissues, we integrated RNA sequencing data and clinical follow‐up information from the TCGA database, encompassing 33 types of cancer. The expression data were normalized through log2 transformation for consistency. Utilizing the median expression value of FOXM1, we categorized HCC patients into high and low expression groups based on the TCGA‐LIHC database. Box plots and paired dot plots were generated using R software (version 3.5.1) for comprehensive visualization. Moreover, to validate FOXM1 protein expression in HCC tissues, we conducted immunohistochemical (IHC) staining with CAB017832 antibody, as per the human protein atlas database online (HPA, https://www.proteinatlas.org).

### 2.3 Correlation between FOXM1 expression and survival prognosis in HCC

Kaplan–Meier (KM) survival curves were employed to investigate the relationship between FOXM1 mRNA expression and survival outcomes. The analysis was conducted using R packages ‘survival’, ‘limma’ and ‘ggpubr’. Additionally, the R package ‘survivalROC’ was utilized to calculate receiver operating characteristic (ROC) curves and associated area under the curve (AUC) values. To further explore the factors influencing the prognosis of HCC patients, univariate and multivariate COX regression models were applied. A *p*‐value of less than 0.05 was considered statistically significant in the screening of factors with a significant predictive effect on HCC patient prognosis.

### 2.4 Correlation between FOXM1 expression and clinicopathological variables in HCC

To assess the associations between FOXM1 expression and clinicopathological variables in HCC, we employed both the Wilcoxon signed‐rank test and logistic regression analysis.

### 2.5 Integration of Protein–Protein Interaction (PPI) network, Coexpressed Genes and Gene Set Enrichment Analysis (GSEA)

To investigate the interactions between FOXM1 and other proteins, a PPI network analysis was conducted using the online STRING website (https://string‐db.org). The coexpression of genes with FOXM1 was examined using Spearman's rank correlation coefficient. For functional enrichment analysis, the gene set ‘c2.cp.kegg.v6.2.symbols’ was obtained from the Molecular Signatures Database (MSigDB). GSEA was performed to identify significant biological pathways and processes with NES (Normalized Enrichment Score) >1.5 and *p* < 0.05.

### 2.6 Molecular docking analysis

In this study, molecular docking was utilized to investigate the interaction between FOXM1 and β‐sitosterol. The 3D structure of FOXM1 (PDB ID: 7FJ2) was obtained in PDB format from the RCSB PDB (https://www.rcsb.org/) database, while the mol2 format file of the 3D structure of β‐sitosterol was acquired from the TCMSP database. Molecular docking of FOXM1 and β‐sitosterol was conducted using PyMoL 2.3.0 and AutoDock. The evaluation of binding activity was based on the minimum binding efficiency, providing insights into the potential interaction strength and stability between FOXM1 and β‐sitosterol.

### 2.7 Cell culture

HCC cell lines (Bel7402, Huh7, HepG2 and SMMC7221), along with the LO2 cell line, were generously provided by Dr. Chuanlong Zhu from the Department of Infectious Disease, Jiangsu Provincial People's Hospital. These cell lines were cultured in Dulbecco's Modified Eagle Medium (DMEM, Gibco, USA), supplemented with 1% penicillin/streptomycin and 10% fetal bovine serum (FBS, Gibco, USA). Maintained in a 37°C incubator with 5% CO_2_, cells from the third to sixth generation were utilized in subsequent experiments. For β‐sitosterol treatment, the compound was dissolved in 100% ethanol, resulting in a final medium ethanol concentration of 0.5%. Control cells were supplemented with medium containing the same concentration of ethanol (0.5%) as the cultures treated with β‐sitosterol. For the treatment with CHIR‐99021 (Selleck, USA), dissolve the compound in DMSO and store it at −20°C until ready for use.

### 2.8 Generation of FOXM1 overexpression cell lines

To achieve FOXM1 overexpression, the cDNAs of FOXM1 were successfully cloned into lentivirus‐based vectors (GeneChem, China) following the manufacturer's protocols. Lentivirus was then diluted and mixed with HitransG A as per the manufacturer's instructions. The lentivirus particles carrying FOXM1 cDNAs were then used to transfect the HCC cells. Transfected cells were subjected to selection with 5 μg/mL puromycin for 1 week to ensure stable integration of the lentiviral construct. The efficiency of FOXM1 overexpression was evaluated by western blotting, confirming the successful establishment of FOXM1 overexpression cell lines.

### 2.9 Cell proliferation assay

To evaluate the cytotoxicity of β‐sitosterol on LO2 cells and its impact on the proliferation of HCC cell lines, a Cell Counting kit‐8 (CCK‐8) assay (Beyotime, China) was employed. At specific time points, the culture medium in each well was replaced with a medium containing 10 μL of WST‐8 solution. The samples were then incubated in a 5% CO_2_ incubator at 37°C for 2 h. Following incubation, the absorbance was measured at 450 nm using a microplate reader.

### 2.10 Assessment of cell migration and invasion

The scratch assay was employed to evaluate the effect of β‐sitosterol on HCC cell migration. HCC cells were seeded in six‐well plates and allowed to reach 90% confluence. Linear scratches were created using a standard 200 μL pipette, followed by incubation in FBS‐free medium for 48 h. The extent of wound closure was observed and imaged using a microscope.

For investigating the effect of β‐sitosterol on HCC cell invasion, the transwell assay (8 μm pore size; Corning, China) was utilized. The cell density was adjusted to 1 × 10^5^, and 200 μL of cell suspension along with various concentrations of β‐sitosterol was added to the upper chamber of 24‐well transwell plates. The lower chamber was filled with 500 μL of medium containing 10% FBS. After 24 h of incubation at 37°C, the cells that had invaded through the membrane were fixed in 4% paraformaldehyde and stained with 4′,6‐diamidino‐2‐phenylindole. Subsequently, the stained cells were counted under a fluorescence microscope.

### 2.11 Real‐time PCR (qPCR)

In this study, qPCR was utilized to examine the expression levels of FOXM1 and GAPDH in both HCC cells and LO2 cells. Total cellular RNA was extracted using the RNAprep Pure Cell Kit (TianGen, China), and subsequently, reverse transcription was performed to synthesize cDNA using the TIANscript RT Kit (TianGen, China). The qPCR analysis was conducted using the QPCR PreMix (SYBR Green) Kit (TianGen, China), and the relative gene expression levels were calculated using the $2^{-\Delta \Delta C_{t}}$ method. Primer sequences used for qPCR were as follows: FOXM1, 5′‐CTATACGTGGATTGAGGACC‐3′ (Forward) and 5′‐CTATACGTGGATTGAGGACC‐3′ (Reverse); GAPDH, 5′‐GACCTGACCTGCCGTCTAG‐3′ (Forward) and 5′‐GACCTGACCTGCCGTCTAG‐3′ (Reverse).

### 2.12 Western blot analysis

The expression levels of all proteins involved in this study were examined using western blot analysis. First, cells were lysed using a radioimmunoprecipitation buffer fortified with 1 mM PMSF and a protease inhibitor (both from Beyotime, China). The lysates were kept on ice for 30 min to ensure complete lysis. The total protein concentration in the lysates was determined using the BCA protein assay kit (Beyotime, China). The proteins in the samples were then separated via 10% SDS‐PAGE and subsequently transferred onto a PVDF membrane. Utilizing the prestained colour protein ladder (Beyotime, China) as a guide, the membranes were horizontally cleaved to obtain the target protein bands. The membranes were then treated with a blocking solution (Beyotime, China) at room temperature for 20 min to prevent non‐specific antibody binding. This was followed by overnight incubation with the primary antibodies (Abcam, USA) at 4°C, promoting specific binding to the target proteins. Finally, the protein bands on the membranes were visualized using the Odyssey® Dlx Imaging System.

### 2.13 In vivo animal studies

Approval for the animal studies was obtained from the Ethics Committee of Hainan Medical University (Permit Number: HYLL‐2022‐384), and all procedures followed the ethical guidelines set by the National Institutes of Health for animal use. Female BALB/C nude mice, 5 weeks old, were procured from Shenyang Wan Class Biotechnology Co., Ltd (China). Subcutaneous implantation of 1 × 10^7^ HepG2 cells into the right dorsal flank of the mice‐initiated tumour growth, and tumour volume and weight were recorded every 5 days. The average tumour volume was calculated using the formula: Tumour volume = (length × width^2^)/2. For the β‐sitosterol treatment group, mice were intraperitoneally injected with β‐sitosterol at various doses (50, 100 or 200 mg/kg) for 28 days, while the control group received an equivalent volume of normal saline injections. At the end of the study period, all animals were euthanized, and their tumours were measured and imaged.

In addition, a HepG2‐Luc cell line was established, characterized by stable expression of luciferase. These cells were injected into nude mice via the tail vein at a concentration of 2 × 10^6^ cells. Similar to the subcutaneous model, the β‐sitosterol treatment group received intraperitoneal injections of β‐sitosterol at varying doses (50, 100 or 200 mg/kg) for 28 days, while the control group received normal saline injections. After the treatment period, in vivo imaging was employed to observe the migration of HCC cells in the mice. Subsequently, the mice were euthanized, and their liver tissues were collected, weighed and photographed.

### 2.14 IHC

To assess FOXM1 expression in tumour tissues, the samples were fixed in formalin and subsequently embedded in paraffin. Deparaffinization was carried out through two consecutive 5 min washes with fresh xylene, followed by rehydration in graded ethanol baths. To block endogenous peroxidase activity, the tissue sections were treated with a 3% hydrogen peroxide buffer for 5 min. Non‐specific staining was further prevented by blocking with 1% goat serum in PBS. The tissue sections were then incubated with the FOXM1 antibody (Affinity, DF6962) at 4°C overnight. Subsequently, a secondary rabbit antibody was applied at room temperature for 1 h. Finally, the IHC staining was visualized using DAB (3,3′‐diaminobenzidine), allowing for the visualization and localization of FOXM1 expression in the HCC tissues.

### 2.15 Statistical analysis

All data were expressed as mean ± standard deviation (SD) from three independently performed assays. Statistical analyses were performed using GraphPad Prism 9 software (GraphPad Software, San Diego, CA, USA). For multiple group comparisons, one‐way anova with Tukey's or Dunnett's post hoc tests were employed to identify significant differences among the groups. A *p*‐value of less than 0.05 (*p* < 0.05) was considered statistically significant. For comparisons between two groups, a student's *t*‐test was utilized in generating box plots and paired dot plots.

## 3 RESULTS

### 3.1 Effect of β‐sitosterol on LO2 cell viability

**FIGURE 2:**
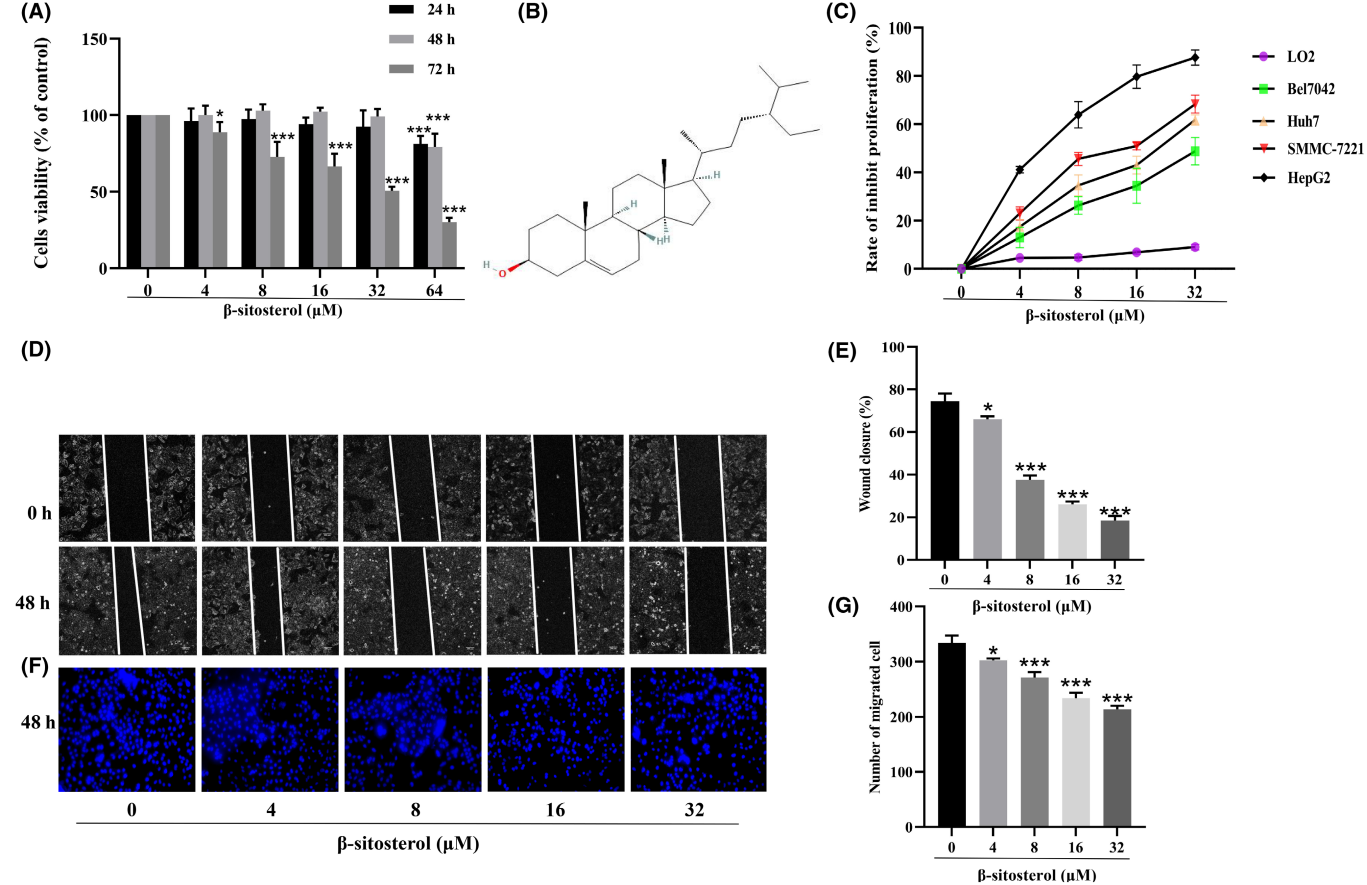
Treatment with 4, 8, 16 and 32 μM β‐sitosterol for 48 h showed no effect on the viability of LO2 cells; nevertheless, it significantly impeded the proliferation and migration of HepG2 cells. (A) The effect of β‐sitosterol on LO2 cell viability was examined using the CCK‐8 assay. (B) The molecular structural formula of β‐sitosterol. (C) The effect of β‐sitosterol on HCC cell proliferation was investigated. (D) The scratch assay was used to detect the effect of β‐sitosterol on HepG2 cell migration. (E) Statistical histogram of the wound closure rate. (F) The transwell assay was used to detect the effect of β‐sitosterol on HepG2 cell invasion. (G) Statistical histogram showing the number of migrating cells. Statistical analyses were conducted through one‐way anova with Dunnett's post hoc test. *p < 0.05, **p < 0.01, ***p < 0.001 versus Control group (without treatment with β‐sitosterol).

We aimed to explore the potential toxicity of β‐sitosterol on normal hepatocytes using the LO2 cell line as our model system. We conducted cell viability assays on LO2 cells treated with various concentrations (4, 8, 16, 32 and 64 μM) of β‐sitosterol for different time durations (24, 48 and 72 h). The results indicated that concentrations of 4, 8, 16 and 32 μM β‐sitosterol, when administered for 24 and 48 h, had no significant impact on cell viability (Figure 2A). However, when LO2 cells were treated with 64 μM β‐sitosterol for 24, 48 and 72 h, a significant inhibitory effect on cell viability was observed. Additionally, treatment with 4, 8, 16 and 32 μM β‐sitosterol for 72 h also resulted in decreased cell viability. Based on these findings, we selected the 48‐h time point as the treatment duration for further investigation of β‐sitosterol's effects on LO2 cells.

### 3.2 β‐Sitosterol inhibits the proliferation, migration and invasion of HCC cells

We investigated the role of β‐sitosterol in HCC cells by assessing its effects on cell proliferation at different concentrations (4, 8, 16 and 32 μM) in various HCC cell lines (Bel7402, Huh7, HepG2 and SMMC7221), as well as in LO2 cells, using a CCK8 assay. The results presented in Figure 2C revealed that β‐sitosterol significantly reduced the proliferation index of HCC cells (Bel7402, Huh7, HepG2 and SMMC7221) in a dose‐dependent manner. Notably, HepG2 cells exhibited the most considerable inhibitory response to β‐sitosterol, prompting us to choose HepG2 cells for subsequent experiments. To further investigate the effects of β‐sitosterol on HepG2 cell behaviour, we conducted scratch assays and transwell assays to assess cell migration and invasion capacity. The findings demonstrated that HepG2 cells treated with β‐sitosterol exhibited a slower wound closure rate and reduced invasion when compared to untreated HepG2 cells (Figure 2D–G). In conclusion, our study suggests that β‐sitosterol possesses the ability to inhibit the proliferation, migration and invasion of HepG2 cells.

### 3.3 Predicting potential targets of β‐sitosterol against HCC

**FIGURE 3:**
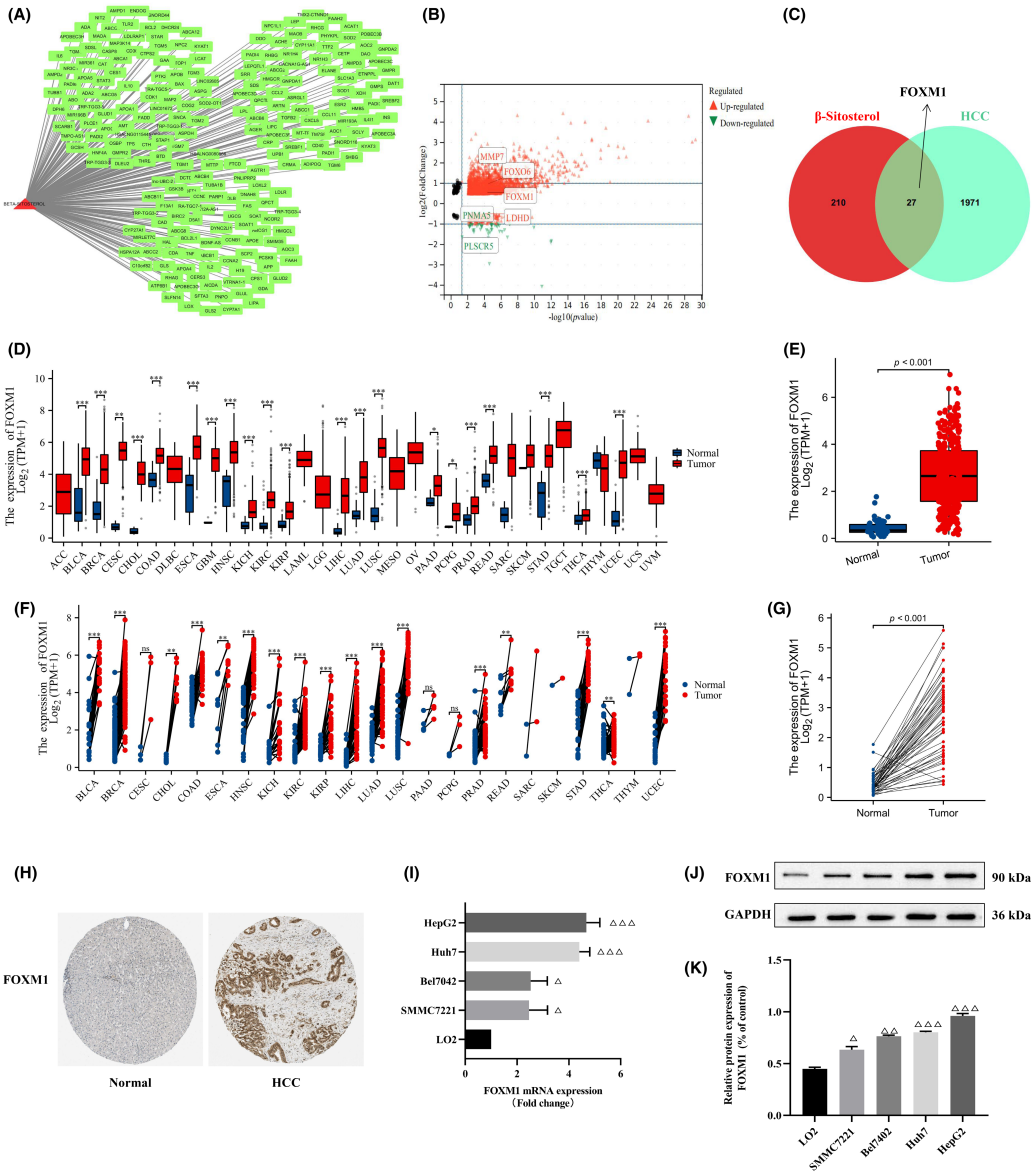
Predicting potential targets of β‐sitosterol against HCC. (A) β‐Sitosterol's potential targets were predicted using GeneCards. (B) HCC‐associated targets were obtained from TCGA database. (C) Venn diagram showing overlapping targets of β‐sitosterol and HCC. (D) FOXM1 expression boxplot across various cancers. (E) Boxplot showing the expression of FOXM1 in HCC. (F) Paired dot plot of FOXM1 expression across multiple cancers. (G) Paired dot plot showing the expression of FOXM1 in HCC. (H) FOXM1 expression in HCC and normal tissues from the HPA database. (I) The mRNA expression of FOXM1 was assessed in HCC cells and LO2 cells using qPCR. (J) The protein expression of FOXM1 was evaluated in HCC cells and LO2 cells using western blot. (K) FOXM1 protein development grey value analysis histogram. Statistical analyses were carried out using one‐way anova with Dunnett's post hoc test. Additionally, box plots and paired dot plots were generated employing a Student's *t*‐test. *p < 0.05, **p < 0.01, ***p < 0.001 versus Normal group or LO2 cell group. ∆*p* < 0.05, ∆∆*p* < 0.01, ∆∆∆*p* < 0.001 versus LO2 cell group.

We aimed to discover potential targets of β‐sitosterol against HCC. Initially, we retrieved β‐sitosterol targets from GeneCards databases, revealing a total of 272 associated targets, including FOXM1, GSK‐3β and BCL2 (Figure 3A). Next, we utilized the TCGA database to identify potential targets specifically relevant to HCC. Our analysis indicated that FXOM1, MMP7 and FOXO6 were upregulated, while PLSCR and PNMA5 were downregulated in HCC (Figure 3B). Finally, to determine the potential targets of β‐sitosterol in the context of HCC, we compared the potential targets of HCC with those of β‐sitosterol. Remarkably, we found that FOXM1 emerged as a common target shared by both HCC and β‐sitosterol (Figure 3C). According to our research findings, it can be concluded that FOXM1 is a potential target of β‐sitosterol in its anti‐HCC effects.

### 3.4 FOXM1 overexpression in HCC tissues and cell lines

To explore the expression level of FOXM1 in HCC, we conducted a comprehensive analysis using the TCGA database, evaluating FOXM1 expression in various tumour types. Our findings revealed a significant upregulation of FOXM1 in numerous cancers, including bladder cancer, breast cancer, cervical cancer, bile duct cancer, colon cancer, oesophageal cancer, head and neck cancer, kidney chromophobe, kidney clear cell carcinoma, kidney papillary cell carcinoma, HCC, lung adenocarcinoma, lung squamous cell carcinoma, prostate cancer, rectal cancer and stomach cancer (Figure 3D,E). Moreover, our investigation specifically focused on HCC, where we observed a noteworthy observation: FOXM1 expression was significantly stronger in HCC tissue compared to normal liver tissue (Figure 3F,G). To further validate these findings, we utilized the HPA database and found that the HCC tissue sample stained highly for FOXM1 (CAB017832), while no staining was observed in the normal adjacent liver tissue sample (Figure 3H). To strengthen our understanding of FOXM1's involvement in HCC, we analysed its expression in HCC cell lines and non‐cancerous LO2 cells through qPCR and western blot. The results consistently demonstrated a significant upregulation of FOXM1 mRNA and protein in HCC cell lines when compared to LO2 cells (Figure 3I–K).

### 3.5 Prognostic implications of FOXM1 upregulation in HCC

**FIGURE 4:**
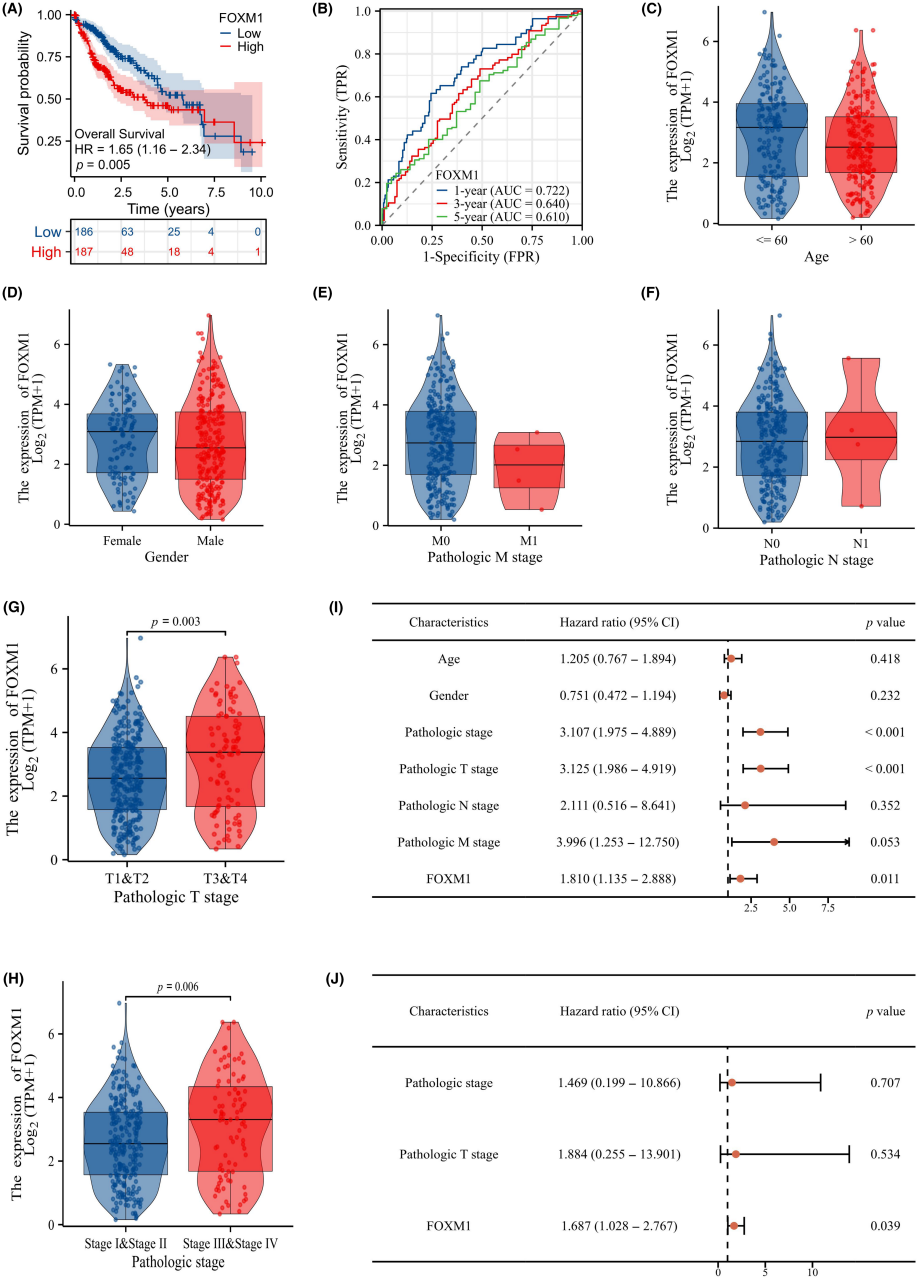
High FOXM1 expression is linked to poor prognosis in HCC patients. (A) The KM method, along with the log‐rank test, was employed to compare the overall survival rates among HCC patients exhibiting low and high FOXM1 expression. (B) ROC curves of FOXM1 associated with 1‐, 3‐ and 5‐year AUCs. (C–H) The relationship between FOXM1 expression and age, sex and stage of T, N, M was analysed using the Wilcoxon rank‐sum test and logistic regression. (I) Univariate Cox hazard regression analysis. (J) Multivariate Cox hazard regression analysis.

After evaluating FOXM1 expression in HCC tissues and cell lines, we investigated its association with patient outcomes using clinical data sets from the TCGA database. KM survival analysis revealed that low FOXM1 expression was linked to longer overall survival in HCC patients, based on median FOXM1 expression (Figure 4A). Additionally, ROC curve analysis demonstrated promising predictive accuracy for 1‐, 3‐ and 5‐year overall survival with AUCs of 0.772, 0.640 and 0.610 respectively (Figure 4B). Further analyses explored the relationships between FOXM1 expression and various clinicopathological variables. The Wilcoxon signed‐rank test and logistic regression revealed significant correlations between FOXM1 expression and pathologic T stage and pathologic stage (Figure 4C–H). Univariate and multivariate Cox hazard regression analyses were conducted to identify independent prognostic factors for HCC. Univariate analysis showed significant associations between overall survival and pathologic stage, pathologic T stage and FOXM1 expression (Figure 4I). Importantly, multivariate Cox regression analysis demonstrated that FOXM1 expression independently predicts overall survival in HCC (Figure 4J). In conclusion, our findings indicate that FOXM1 serves as an independent prognostic factor for HCC, with potential implications for patient outcomes.

### 3.6 β‐Sitosterol suppresses HepG2 cell proliferation, migration and invasion via FOXM1 inhibition

**FIGURE 5:**
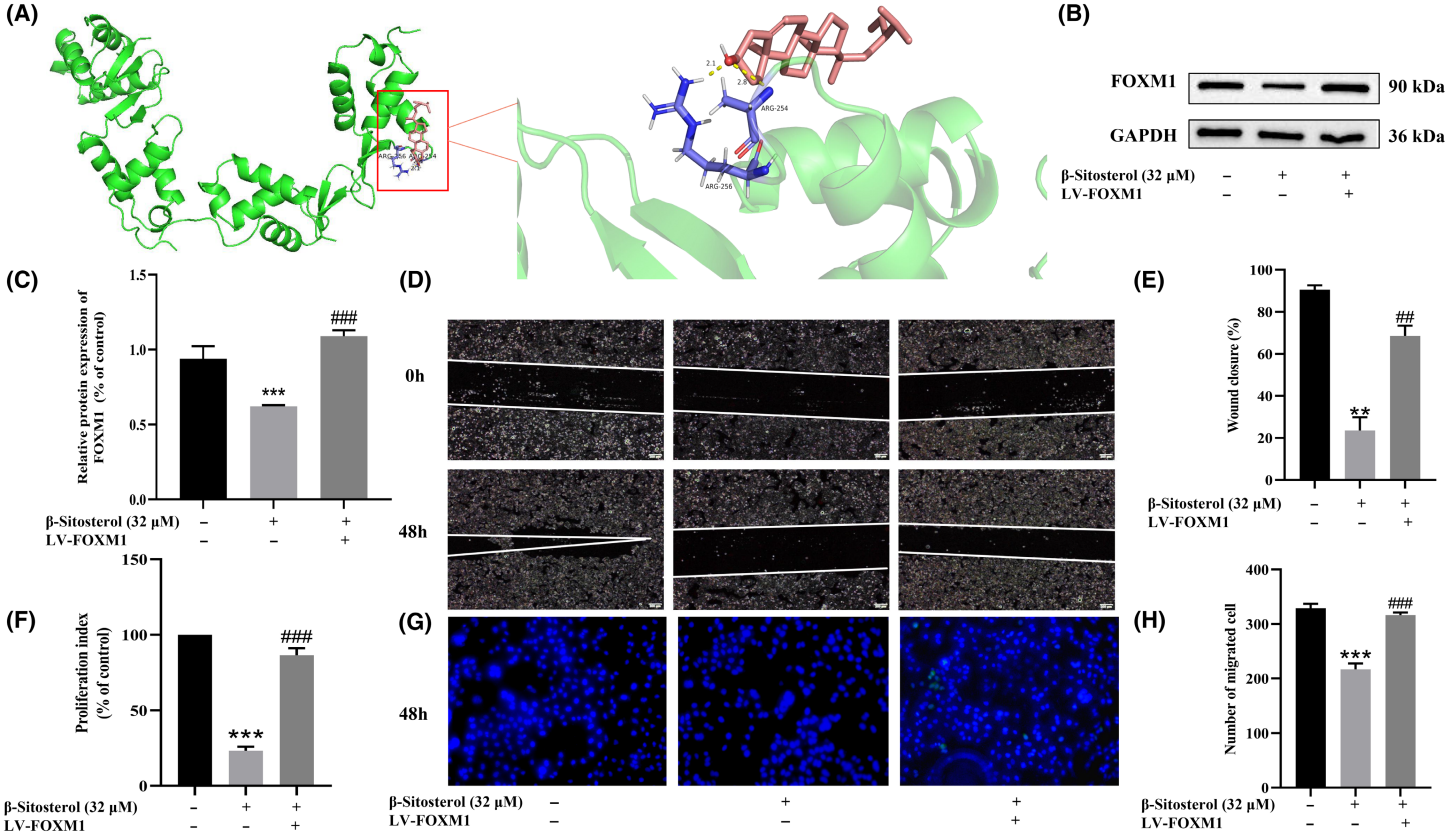
β‐Sitosterol suppresses HepG2 cell proliferation, migration and invasion via FOXM1 inhibition. (A) β‐Sitosterol molecular docking with FOXM1. (B) Western blot was used to detect the effect of β‐sitosterol on FOXM1 expression in HepG2 cells. (C) FOXM1 protein development grey value analysis histogram. (D) The effect of β‐sitosterol on HepG2 cell migration after FOXM1 overexpression was detected. (E) Statistical histogram of the wound closure rate. (F) The effect of β‐sitosterol on HepG2 cell proliferation was examined after FOXM1 overexpression. (G) The effect of β‐sitosterol on HepG2 cell invasion was evaluated after overexpression of FOXM1. (H) Statistical histogram showing the number of migrating cells. Statistical analyses were carried out using one‐way anova with Tukey's post hoc test. *p < 0.05, **p < 0.01, ***p < 0.001 versus Control group (without treatment with β‐sitosterol). #*p* < 0.05, ##*p* < 0.01, ###*p* < 0.001 versus β‐sitosterol treatment group.

We employed molecular docking to assess the interaction between FOXM1 and β‐sitosterol. The results revealed a strong binding capacity between the two, with a binding energy of −13.6 kJ/mol, primarily driven by hydrogen bonding (Figure 5A). Subsequently, western blot analysis demonstrated a significant decrease in FOXM1 expression in HepG2 cells treated with β‐sitosterol compared to DMEM‐treated cells (Figure 5B,C). To further explore the impact of FOXM1 on the proliferation, migration and invasion of HepG2 cells, we used LV‐FOXM lentivirus to overexpress FOXM1 in HepG2 cells. The CCK8, scratch and transwell assays indicated that the upregulation of FOXM1 counteracted the inhibitory effects of β‐sitosterol on proliferation, migration and invasion in HepG2 cells (Figure 5D–H). In conclusion, these findings suggest that β‐sitosterol exerts its inhibitory effects on the proliferation, migration and invasion of HepG2 cells through the downregulation of FOXM1 expression.

### 3.7 β‐Sitosterol suppresses HepG2 cell EMT via FOXM1 inhibition

**FIGURE 6:**
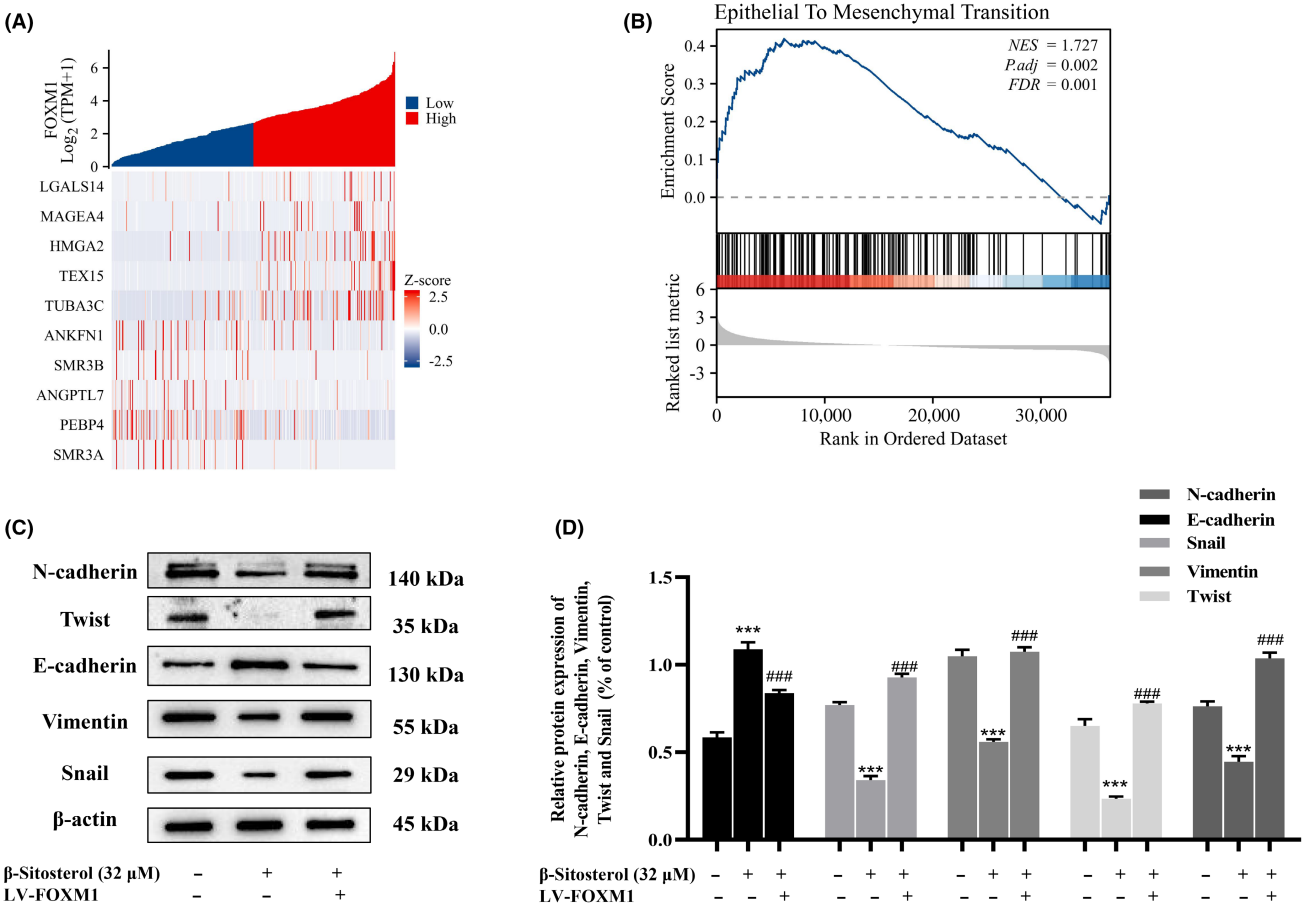
β‐Sitosterol suppresses HepG2 cell EMT via FOXM1 inhibition. (A) Heat map of genes coexpressed with FOXM1. (B) GSEA analysis. (C) The effect of β‐sitosterol on the expression of E‐cadherin, N‐cadherin, Vimentin, Snail and Twist in HepG2 cells was detected after FOXM1 overexpression. (D) E‐cadherin, N‐cadherin, Vimentin, Snail and Twist protein development grey value analysis histogram. Statistical analyses were carried out using one‐way anova with Tukey's post hoc test. *p < 0.05, **p < 0.01, ***p < 0.001 versus. Control group (without treatment with β‐sitosterol). #*p* < 0.05, ##*p* < 0.01, ###*p* < 0.001 versus β‐sitosterol treatment group.

Previous bioinformatics analysis suggested the potential involvement of FOXM1 in HCC metastasis. To confirm FOXM1's role in HCC, we conducted Spearman's rank correlation coefficient analysis, identifying coexpressed genes in HCC. Notably, LGALS14, MAGEA4, HMGA2, SMR3B and PEBP4 showed significant associations with FOXM1 expression in HCC (Figure 6A). GSEA analysis further revealed that EMT was differentially enriched in the positively correlated phenotype with FOXM1 expression (Figure 6B). Given that EMT is a critical process for cancer cell migration and invasion, we investigated whether β‐sitosterol and FOXM1 play roles in EMT regulation in HCC. Our findings demonstrated that β‐sitosterol downregulated N‐cadherin, Twist, Vimentin and Snail expression while upregulating E‐cadherin (Figure 6C,D). Conversely, the overexpression of FOXM1 resulted in adverse effects. These results collectively indicate that β‐sitosterol inhibits EMT in HepG2 cells by downregulating FOXM1 expression, leading to the suppression of migration and invasion in HepG2 cells.

### 3.8 β‐Sitosterol suppresses the Wnt/β‐catenin signalling pathway via FOXM1 inhibition in HepG2 cells

**FIGURE 7:**
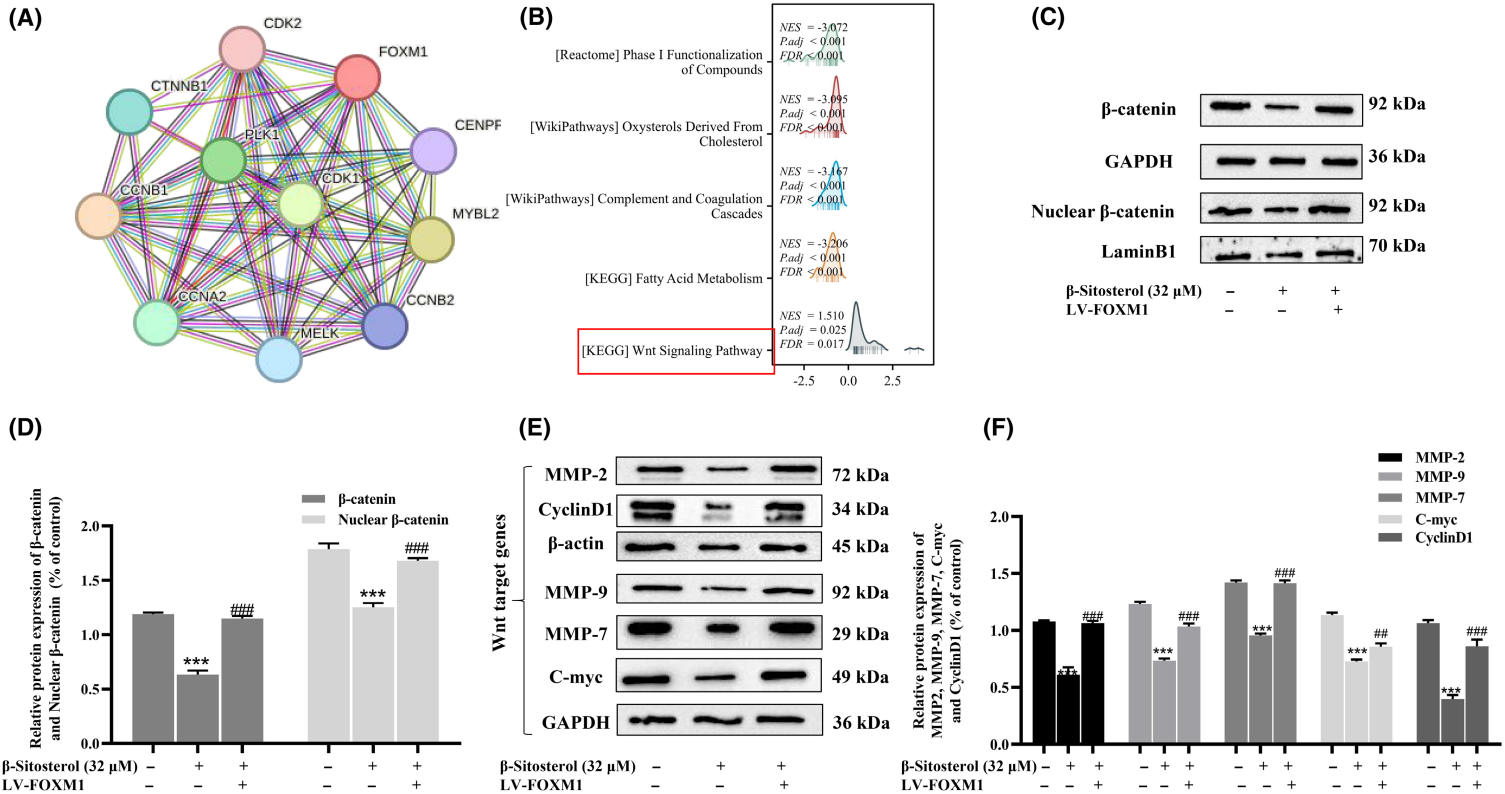
β‐Sitosterol Suppresses the Wnt/β‐Catenin Signalling Pathway via FOXM1 Inhibition in HepG2 Cells. (A) FOXM1‐related PPI network. (B) GSEA analysis. (C) The effect of β‐sitosterol on the expression of total β‐catenin and nuclear β‐catenin in HepG2 cells was examined after FOXM1 overexpression. (D) β‐catenin and nuclear β‐catenin protein development grey value analysis histogram. (E) The effect of β‐sitosterol on the expression of Wnt target genes (MMP2, MMP7, MMP9, C‐myc and CyclinD1) was examined in HepG2 cells after FOXM1 overexpression. (F) MMP2, MMP7, MMP9, C‐myc and CyclinD1 protein development grey value analysis histogram. Statistical analyses were carried out using one‐way anova with Tukey's post hoc test. *p < 0.05, **p < 0.01, ***p < 0.001 versus Control group (without treatment with β‐sitosterol). #*p* < 0.05, ##*p* < 0.01, ###*p* < 0.001 versus β‐sitosterol treatment group.

To further elucidate the roles of FOXM1 in HCC, we utilized the STRING database to identify proteins associated with FOXM1. Our analysis revealed significant associations between FOXM1 and several proteins, including CTNNB1, CDK2, CCNB1, CCNA2 and MELK (Figure 7A). Additional GSEA analysis highlighted the differential enrichment of the Wnt signalling pathway in the phenotype positively correlated with FOXM1 expression (Figure 7B). The canonical Wnt/β‐Catenin signalling pathway is stimulated by the translocation of accumulated β‐catenin from the cytoplasm to the nucleus.^23^ In light of this, we investigated the protein expressions of total and nuclear β‐catenin. Western blot analysis showed that β‐sitosterol effectively inhibited total and nuclear β‐catenin protein expression, an effect that was reversed by FOXM1 overexpression (Figure 7C,D). We further explored the regulatory effect of β‐sitosterol and FOXM1 on β‐catenin by examining the expression of downstream target genes in the Wnt/β‐catenin signalling pathway (C‐myc, CyclinD1, MMP2, MMP7 and MMP9). β‐Sitosterol significantly downregulated the expression of C‐myc, Cyclin D1, MMP2, MMP7 and MMP9 compared to the control group, while FOXM1 overexpression had the opposite effects (Figure 7E,F). In summary, these findings suggest that β‐sitosterol may attenuate the progression and malignancy of HCC by suppressing the activation of the Wnt/β‐catenin signalling pathway via FOXM1 inhibition.

### 3.9 Counteraction of β‐sitosterol's inhibitory effects on HepG2 cell proliferation, migration and invasion by Wnt/β‐catenin pathway activation

**FIGURE 8:**
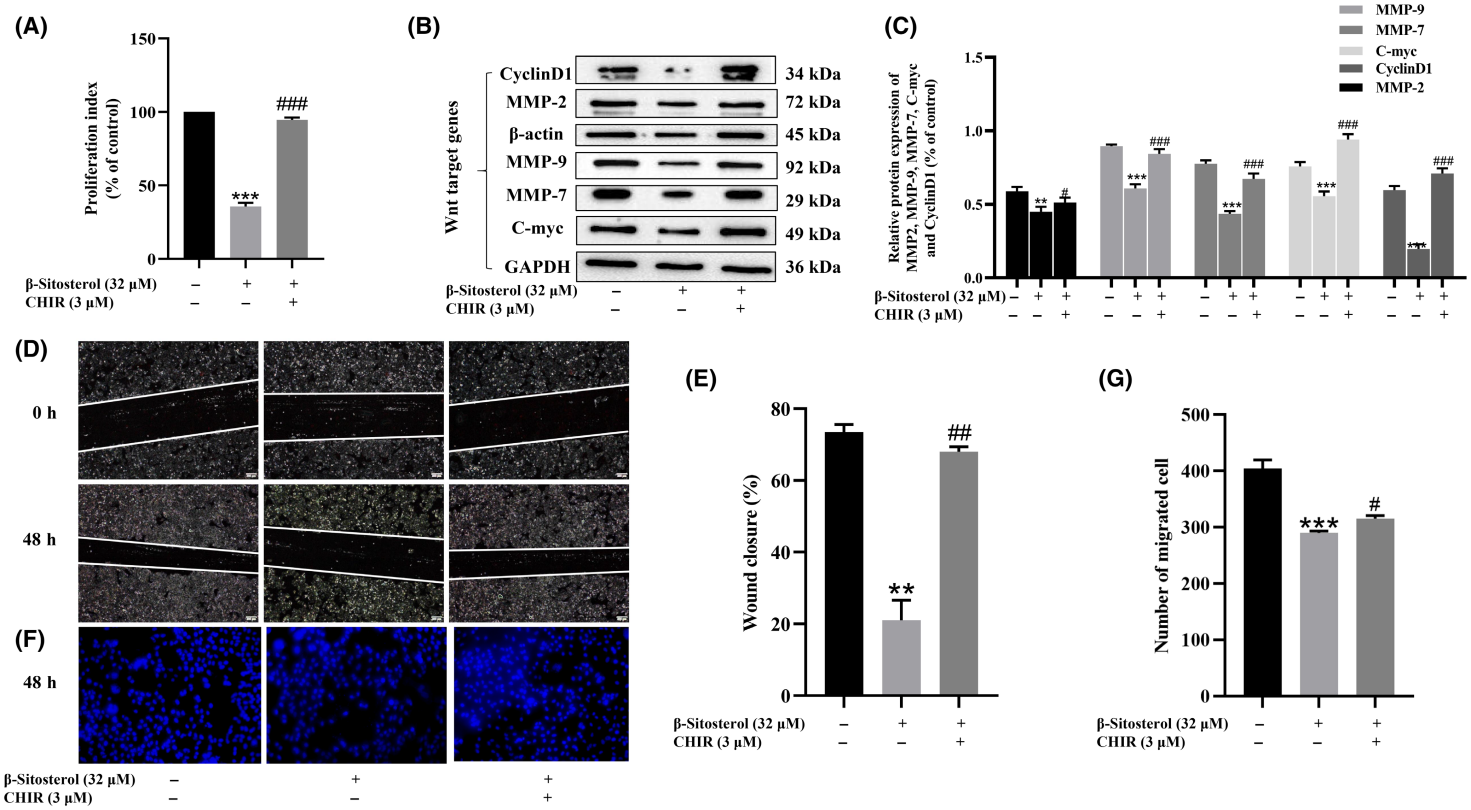
A counteraction of β‐sitosterol's inhibitory effects on HepG2 cell proliferation, migration and invasion by Wnt/β‐Catenin pathway activation. (A) The effect of β‐sitosterol on HepG2 cell proliferation was detected after treatment with CHIR‐99021. (B) The effect of β‐sitosterol on the expression of Wnt target genes (MMP2, MMP7, MMP9, C‐myc and CyclinD1) was evaluated after treatment with CHIR‐99021. (C) MMP2, MMP7, MMP9, C‐myc and CyclinD1 protein development grey value analysis histogram. (D) The effect of β‐sitosterol on the migration of HepG2 cells after treatment with CHIR‐99021 was observed. (E) Statistical histogram of the wound closure rate. (F) The effect of β‐sitosterol on the invasion of HepG2 cells was evaluated after treatment with CHIR‐99021. (G) Statistical histogram showing the number of migrating cells. Statistical analyses were carried out using one‐way anova with Tukey's post hoc test. *p < 0.05, **p < 0.01, ***p < 0.001 versus Control group (without treatment with β‐sitosterol). #*p* < 0.05, ##*p* < 0.01, ###*p* < 0.001 versus β‐sitosterol treatment group.

To further validate the importance of Wnt/β‐catenin signalling in the inhibitory effect of β‐sitosterol on HCC, we conducted rescue experiments using CHIR‐99021, a Wnt/β‐catenin signalling agonist. The CCK‐8 assays demonstrated a significant restoration of the proliferation inhibition ability in HepG2 cells treated with β‐sitosterol upon subsequent treatment with CHIR‐99021 (Figure 8A). Additionally, transwell and scratch assays revealed that the migration and invasion of HepG2 cells, suppressed by β‐sitosterol treatment, were reversed by CHIR‐99021 treatment (Figure 8D–G). Moreover, the expression of C‐myc, Cyclin D1, MMP2, MMP7 and MMP9 was upregulated in β‐sitosterol‐treated HepG2 cells after treatment with CHIR‐99021 (Figure 8B,C). These findings collectively confirm that β‐sitosterol effectively inhibits the proliferation, migration and invasion of HepG2 cells by impeding the Wnt/β‐Catenin signalling pathway.

### 3.10 β‐Sitosterol inhibits HCC xenograft growth and metastasis through FOXM1 suppression

**FIGURE 9:**
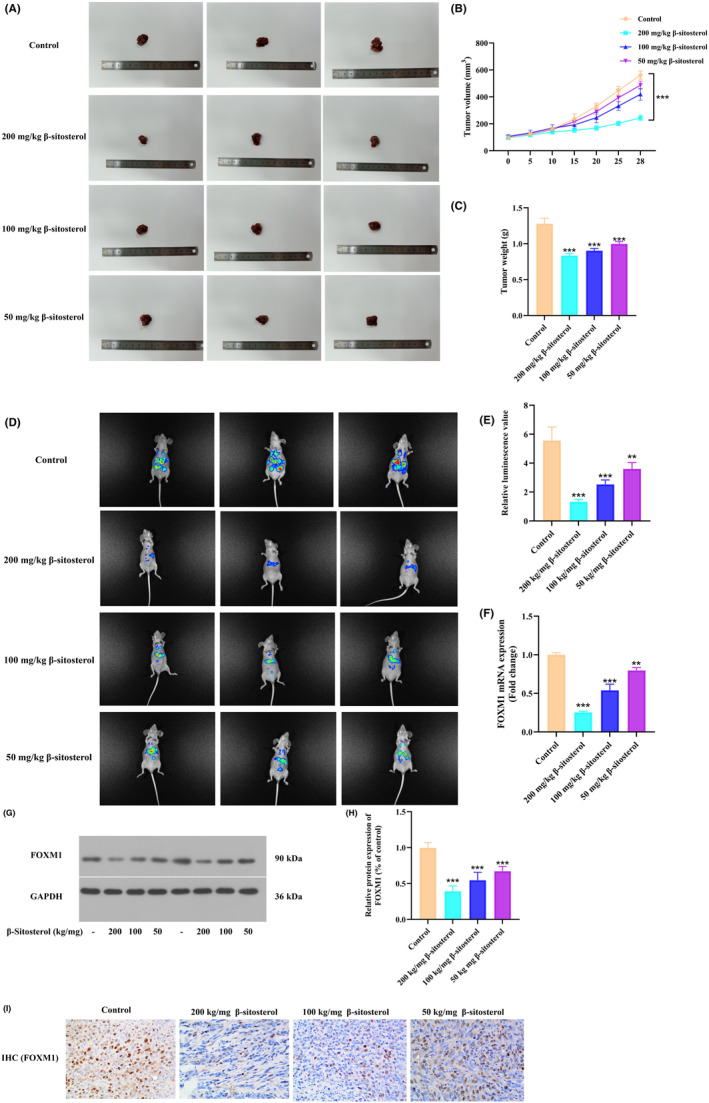
β‐Sitosterol inhibits HCC xenograft growth and metastasis through FOXM1 suppression. (A) Pictures of tumours in mice treated with β‐sitosterol and control mice. (B) Tumour volumes were measured every 5 days after injection. (C) Tumour weights were measured. (D) In vivo imaging was performed after tail vein injection. (E) Statistical histogram displaying the relative values of luminescence. (F) The expression of FOXM1 mRNA was detected using qPCR. (G) The protein expression of FOXM1 was evaluated by western blot. (H) FOXM1 protein development grey value analysis histogram. (I) Immunofluorescence was performed for FOXM1 (original magnification × 400). Statistical analyses were carried out using one‐way anova with Dunnett's post hoc test. *p < 0.05, **p < 0.01, ***p < 0.001 versus Control group (without treatment with β‐sitosterol).

Following in vitro experiments confirming the effects of β‐sitosterol on proliferation, migration and invasion, we investigated its role in cancer formation in vivo using a xenograft mouse model. Subcutaneous implantation of HepG2 cells in the right flank of the animals revealed that β‐sitosterol significantly reduced tumour growth rates, resulting in a dramatic reduction in tumour volume (Figure 9B). Tumours were excised 28 days after inoculation, and images demonstrated lighter tumour weights in the β‐sitosterol group compared to the control group (Figure 9A,C). To explore the impact of β‐sitosterol on HCC metastasis, the same cells were injected into the tail vein of nude mice. In vivo fluorescence imaging indicated that tumour metastasis was inhibited by β‐sitosterol (Figure 9D,E). To gain further insight into the mechanism underlying β‐sitosterol's inhibitory effects on tumour growth and metastasis in vivo, we assessed the expression of FOXM1 in tumour tissues using qPCR, IHC and western blotting. The results demonstrated that β‐sitosterol significantly reduced the mRNA and protein levels of FOXM1 (Figure 9F–I). Consistent with the in vitro study, these findings indicate that β‐sitosterol suppresses the growth and metastasis of HCC cells through FOXM1 in vivo.

## 4 DISCUSSION

β‐Sitosterol, a phytonutrient derived from plants found in various vegetable oils, nuts and herbal remedies,^24^ has been recognized for its anti‐tumour properties.^25^ Previous studies have reported its inhibitory effects on human colon cancer cells by affecting sphingomyelin metabolism and its ability to suppress androgen‐independent and androgen‐dependent prostate cancer cell lines.^26^ Additionally, the use of β‐sitosterol‐assisted silver nanoparticles has shown significant inhibition of HepG2 cell proliferation, promotion of apoptosis and induction of ROS and Nrf‐2 expression.^27^ These studies suggest that β‐sitosterol may have potential inhibitory effects on HCC development. In this study, we investigated the effects of β‐sitosterol on HCC using in vitro and in vivo models. We found that β‐sitosterol can effectively inhibit the proliferation, migration and invasion of HepG2 cells both in vitro and in vivo, revealing its suppressive function in HCC.

To gain insights into the underlying mechanisms of β‐sitosterol's effects on HCC, we conducted an analysis using TCGA and GeneCards databases to identify potential targets. Among the candidates, FOXM1 emerged as a promising target due to its pivotal role in regulating tumorigenesis and cancer progression, as supported by previous studies.^28^, ^29^, ^30^ Specifically, FOXM1 has been shown to promote HCC cell proliferation both in vitro and in vivo through its regulation of KIF4A expression.^14^ Our analysis of clinical samples from TCGA, HPA and ICGC databases consistently revealed significant upregulation of FOXM1 in human HCC samples compared to adjacent normal tissues. Notably, patients with higher FOXM1 expression levels exhibited shorter overall survival, highlighting FOXM1's potential as an independent prognostic factor for HCC according to both univariate and multivariate Cox hazard regression analysis. These findings underscore the critical role of FOXM1 in HCC development and progression. In our experimental study, we observed that β‐sitosterol effectively downregulated FOXM1 expression in both in vitro HepG2 cells and in vivo models. To investigate the impact of FOXM1 in the context of β‐sitosterol's inhibitory effects on HCC progression, we stably overexpressed FOXM1 in HepG2 cells. Notably, the overexpression of FOXM1 was found to counteract the inhibitory effects of β‐sitosterol on HepG2 cell proliferation, migration and invasion. These results collectively provide compelling evidence that β‐sitosterol exerts its inhibitory effects on HCC cells through FOXM1 downregulation.

EMT plays a critical role in various aspects of cancer, including the onset of cancer, development of cancer stemness and malignancy, metastasis, metabolic reprogramming and drug resistance.^31^, ^32^, ^33^ Recent research by Cui et al. demonstrated that ENO3 inhibits the migration and invasion of HCC cells in vitro by suppressing EMT.^34^ In our study, we explored the potential significance of EMT in the context of β‐sitosterol treatment against HCC using GSEA. E‐cadherin, N‐cadherin and Vimentin are widely recognized as primary biomarkers of EMT.^35^, ^36^ Our findings revealed that administration of β‐sitosterol upregulated E‐cadherin expression while downregulating N‐cadherin and Vimentin expression in HepG2 cells. However, this effect was found to be counteracted by FOXM1 overexpression. Moreover, we examined EMT‐activated transcription factors, including Snail and Twist, and observed that β‐sitosterol treatment led to their downregulation in HepG2 cells. Importantly, overexpression of FOXM1 negated the inhibitory effect of β‐sitosterol on Snail and Twist in HepG2 cells. Based on these findings, it appears that β‐sitosterol may act as a suppressor of migration and invasion by inhibiting FOXM1‐mediated EMT in HCC cells.

To elucidate the mechanisms underlying β‐sitosterol's inhibitory effects on the proliferation, migration and invasion of HCC cells, we utilized GSEA to identify FXOM1‐related signalling pathways. The GSEA results indicated that the Wnt/β‐catenin signalling pathway might play a significant role in the action of β‐sitosterol against HCC. It is well known that signalling pathways associated with embryogenesis are excessively activated during tumorigenesis.^37^ The Wnt/β‐catenin signalling pathway has been reported to promote various cellular abilities in tumours, including cell cycle development, proliferation, migration and invasiveness.^38^, ^39^, ^40^ In the context of the Wnt/β‐catenin cascade transduction, the transfer of β‐catenin from the cytoplasm to the nucleus is a crucial event. FOXM1 activates the β‐catenin pathway and contributes to renal fibrosis by regulating members of the multi‐Wnt family.^41^ USP28‐mediated stabilization of FOXM1 significantly enhances nuclear β‐catenin transactivation, thereby activating the Wnt/β‐catenin pathway.^42^ Additionally, FOXM1 plays a pivotal role in glioma development by facilitating the nuclear translocation of β‐catenin and augmenting its transcriptional activity in cells.^43^ In our study, we observed that β‐sitosterol reduced the total and nuclear accumulation of β‐catenin, but this effect was counteracted by the overexpression of FOXM1. This nuclear accumulation of β‐catenin leads to the upregulation of downstream Wnt target genes, triggering uncontrollable cellular events. Among these Wnt target genes, C‐myc and Cyclin D1 are associated with cell proliferation, while MMP2, MMP9 and MMP7 are associated with cell migration and invasion.^34^, ^44^ Our findings demonstrated that β‐sitosterol downregulated the expression of C‐myc, Cyclin D1, MMP2, MMP9 and MMP7 in HepG2 cells. Conversely, the overexpression of FOXM1 resulted in the upregulation of these genes. Furthermore, the Wnt/β‐catenin pathway agonist CHIR‐99021 was able to reactivate the proliferation and migration of HepG2 cells that had been inhibited by β‐sitosterol. Based on these results, we have concluded that β‐sitosterol inhibits the growth and metastasis of HCC cells by regulating the Wnt/β‐catenin signalling pathway through FOXM1.

Despite significant advances in diagnostic methods for HCC, the majority of patients are still diagnosed at an advanced stage, limiting therapeutic options.^45^ Current drugs used in clinical practice for HCC have shown poor efficacy and have certain adverse reactions due to the difficulty of reaching the tumour site.^46^, ^47^ Given the ability of the overactivated Wnt/β‐catenin signalling pathway to promote HCC cell proliferation, migration and invasion, β‐sitosterol may serve as a Wnt/β‐catenin axis inhibitor and present a new strategy for treating HCC.

## 5 CONCLUSION

In summary, our study has provided the first evidence that β‐sitosterol plays a critical inhibitory role in HCC growth and metastasis by modulating the Wnt/β‐catenin signalling pathway through FOXM1. These findings collectively suggest that β‐sitosterol holds significant potential as a therapeutic strategy for HCC. However, further in‐depth studies and preclinical trials are essential to validate the safety and efficacy of β‐sitosterol as a potential therapeutic strategy for HCC.

## AUTHOR CONTRIBUTIONS

**Yuankun Chen:** Conceptualization (equal); data curation (equal); formal analysis (equal); funding acquisition (equal); investigation (equal); methodology (equal); writing – review and editing (equal). **Yijun Yang:** Formal analysis (equal); investigation (equal); methodology (equal). **Nengyi Wang:** Formal analysis (equal); investigation (equal); methodology (equal). **Rui Liu:** Investigation (equal); methodology (equal); writing – review and editing (equal). **Qiuping Wu:** Formal analysis (equal); investigation (equal). **Hua Pei:** Conceptualization (equal); methodology (equal); supervision (equal). **Wenting Li:** Conceptualization (equal); funding acquisition (equal); methodology (equal); supervision (equal); writing – review and editing (equal).

## FUNDING INFORMATION

This work was supported by the National Natural Science Foundation of China (No. 81503282, 82260125 and 31500926), Hainan Provincial Natural Science Foundation of China (No. 822MS181, 823RC591 and 822QN473), Key project of Anhui Province (No. S202104j07020097) and Hainan Province Clinical Medical Center.

## CONFLICT OF INTEREST STATEMENT

The authors report no declarations of interest. The authors alone are responsible for the content and writing of the paper.

## Data Availability

The data that support the findings of this study are available from the corresponding author, Wenting Li, upon reasonable request.
